# The development and validation of the Offensive-Type Taijin-Kyofu-Sho Scale (OTKSS): A preliminary study using a sample of Japanese University Students

**DOI:** 10.1371/journal.pmen.0000223

**Published:** 2025-05-28

**Authors:** Ryotaro Ishikawa, Naoki Yoshinaga, Satoshi Asakura, Graham Thew, Kana Endo, Takuma Ishigaki, Yuki Nishiguchi, Akira Aoki

**Affiliations:** 1 Faculty of Clinical Psychology, Taisho University, Toshima, Tokyo; 2 School of Nursing, Faculty of Medicine, University of Miyazaki, Miyazaki, Japan; 3 Health Care Center, Hokkaido University, Sapporo, Japan; 4 Department of Experimental Psychology, University of Oxford, Oxford, United Kingdom; 5 Oxford Health NHS Foundation Trust, Oxford, United Kingdom; 6 Graduate School of Arts and Sciences, The University of Tokyo, Tokyo, Japan; 7 Faculty of Education, Chiba University, Chiba, Japan; David Geffen School of Medicine: University of California Los Angeles David Geffen School of Medicine, UNITED STATES OF AMERICA

## Abstract

Taijin-kyofu-sho (TKS) is considered a type of social anxiety disorder. Its subtype, offensive-type TKS (OTKS), is characterized by a fear that one’s own body odor, gaze, facial expressions, and physical defects are socially inappropriate and may cause discomfort to others. Existing self-report measures do not specifically examine the symptoms of OTKS. Therefore, this study aimed to develop a self-report measure for assessing OTKS (Offensive-Type Taijin-Kyofu-Sho Scale: OTKSS). The OTKSS was constructed with four subscales containing seven items each. It was administered to 534 Japanese students who screened positive for social anxiety disorder and reported one or more symptoms of OTKS. The data were subjected to an exploratory factor analysis. Additionally, we conducted a correlation analysis comparing OTKSS with other social anxiety and TKS scales. A second survey examined the test–retest reliability across a 4-week interval with 144 students who screened positive for social anxiety disorder and reported one or more symptoms of OTKS. The results of Survey 1 confirmed the psychometric properties of the OTKSS, including convergent and discriminant validity. Survey 2 confirmed the test–retest reliability. The study indicates that OTKSS is a reliable and valid instrument for assessing OTKS. However, as a limitation, the sample in this study was restricted to Japanese university students. Given that the psychometric work of this paper was conducted only on this population, our findings related to the preliminary evidence of reliability and validity apply to this population only. Future studies using larger sample sizes and clinical samples should test the validity of these measures. In addition, an English version of the scale was simultaneously prepared and will be tested in future studies.

## Introduction

Social anxiety is a fast-growing phenomenon that affects youth disproportionately [1,2]. Social Anxiety Disorder (SAD) is characterized by a marked and intense fear of social situations wherein one might be critically observed by others, leading to avoidance of such situations [3]. In the United States, lifetime prevalence and median age of onset of SAD are 12.1% and 13 years, respectively [4]; additionally, the 12-month prevalence estimate is 2.4% across countries from Africa, the Americas, Eastern and Western Europe, Western Pacific, and Eastern Mediterranean [5]. The World Mental Health Japan survey estimated a 12-month prevalence of 0.8% among Japanese people [6]. According to the cognitive-behavioral model of SAD [7], anxious and physical reactions to social situations are maintained by cognitive (e.g., automatic thoughts, negative self-image, and self-focused attention) and behavioral factors (e.g., avoidance and safety behaviors). Safety behaviors are overt or covert acts intended to prevent a feared situation or minimize its consequences [7–9], reduce related distress, or “hide” one’s anxiety (e.g., Voncken et al. [10]). Although temporarily useful, safety behaviors can lead to the non-occurrence of a feared catastrophe being misattributed to the use of such behaviors, consequently reinforcing belief in their preventative function [9,11]. Some safety behaviors have the paradoxical effect of increasing the visibility of somatic anxiety symptoms, thereby increasing the likelihood of the feared outcome [7]. Furthermore, they increase self-focused attention, which decreases the awareness of external cues while heightening that of anxiety symptoms. Self-focused attention results in a greater sense of threat and social incompetency, preventing the refutation of the feared outcome [7]. Therefore, using safety behaviors can reinforce conviction in distorted cognitive biases and maintain anxiety-related distress [11–14].

*Taijin-Kyofu-Sho* (TKS), literally fear (*kyofu*) of interpersonal relationships (*taijin*) in Japanese, was first described by Japanese psychiatrist Masatake Morita in the 1930s [15]. TKS is often considered a subtype of SAD specific to East Asian cultures and is classified in the *Diagnostic and Statistical Manual of Mental Disorders, Fifth Edition, Text Revision* (DSM-5-TR) as a culture-bound diagnosis [3]. The fear associated with social anxiety is of personal humiliation, rejection, or embarrassment, whereas that of TKS is of making others uncomfortable (e.g., my gaze may irritate people). Nevertheless, TKS overlaps substantially with SAD despite its cultural underpinnings. Moreover, features of the offensive subtype of TKS have been reported among Western patients with SAD, suggesting that TKS is not as culture-bound as previously believed [16–18].

TKS has been reported as having “sensitive” and “offensive (OTKS)” subtypes [3,19,20]. The sensitive type has many similarities with SAD [16,19,20] and is characterized by the “fear of being looked at (noticed) by others” (e.g., fear of others noticing their sweating, voice trembling, or blushing). The sensitive subtype of TKS is a condition in which “1) the individual feels that his/her own attitudes, behaviors, and physical characteristics are inadequate in social situations; 2) because of these feelings, the individual suffers persistently from emotional reactions, such as shame, embarrassment, anxiety, fear, and is scared and tense in social situations; 3) due to conditions 1 and 2, the individual feels worried that he/she is unable to maintain healthy relationships with others, and he/she may feel unacceptable, despised, and avoided; 4) while the individual attempts to avoid painful, social, and interpersonal situations, he/she feels reluctant to do so” [19, p. 99].

Unlike the sensitive type of TKS, OTKS is characterized by the fear of causing trouble to others in social situations, which is characterized by the fear of embarrassing oneself when performing in front of others. Additionally, in OTKS, the conviction in the belief that one’s physical characteristics discomfit others is sometimes delusional [3,21,22], earning it the label “convinced TKS” [19,23]. A person with this subtype of TKS also suffers from the sensitive TKS conditions. However, in addition to sensitive TKS conditions, “1) the individual feels certain that he/she has a defect in a particular body part or physical characteristics, such as the eyes, body odor, or appearance, 2) due to condition 1, the individual has a conviction that he/she harms other people or gives others unpleasant feelings, 3) also due to condition 1, the individual has a conviction that others always avoid him/her” [19 p. 99].

Nagata et al. [20] investigated the differences between offensive and non-offensive SAD in 139 Japanese patients, finding that the 52 (37%) diagnosed with the offensive subtype showed significantly higher scores than those with the non-offensive one on the Liebowitz Social Anxiety Scale (LSAS). Choy et al. [16] reported a similar rate for the offensive subtype of SAD among Koreans (25/64, 39%); they also reported that, among the participants from the United States who had SAD, 15.5–39.2% of them had OTKS. These results suggest the prevalence of the offensive subtype among clinical populations.

Regarding OTKS in student samples, Tarumi et al. [24] showed that of 111 college students who met the criteria for SAD, 25 (22.5%) fit the symptomatic profile of OTKS. Reuter et al. [25] studied the prevalence of olfactory reference syndrome (ORS) in German students, based on the *International Classification of Diseases 11*^*th*^ *Revision* (ICD-11) criteria. ORS, which is related to the fear of body odor [25], is known to have symptoms overlapping those of OTKS. The results indicated that the prevalence of ORS was 5.5%, suggesting that ORS may be relatively common in university students [25].

### Four types of OTKS

Based on a comprehensive review of the literature [15,16,19,20,26,27–29], OTKS can be divided into four types. The first is the fear of body odor or ORS, which leads individuals to engage in time-consuming rituals aimed at masking or fixing their odor [30–33]. ORS currently appears in the DSM-V-TR under “Other Specified Obsessive Compulsive Disorders” as *Jiko-shu-kyofu*, a variant of TKS characterized by the fear of having an offensive body odor [3]. In a community epidemiological study of TKS in Japan, 8 out of 132 respondents (6.0%) had specific concerns about body odor [34].

The second is inappropriate eye contact (e.g., inappropriate staring or gaze), or *jiko-shisen kyofu* in Japanese, wherein patients are convinced that they are hurting and offending others through their own displeased glances [16,29,35]. Individuals with this type of OTKS believe that their glance brings others discomfort and convince themselves of the accuracy of their belief by interpreting others’ trivial behavior (e.g., coughing, laughing, sniffing, sneezing, and head-turning) as evidence of displeasure. They feel deeply ashamed, demeaned, and unacceptable, and many eventually avoid social situations altogether. Interestingly, McNally et al. [36] reported a case of TKS in a non-Japanese patient—a Black American woman who avoided people because she feared embarrassing them by furtively glancing at their genital areas.

The third concerns inappropriate facial expressions. Afflicted individuals worry that their facial expressions may stiffen in front of others, making them impossible to change, and consequently, offend or make others uncomfortable [16,19,20,27]. Choy et al. [16] indicated that among Korean patients with SAD and OTKS, 44% were concerned that their stiff facial expressions could offend others, a proportion higher than that for other physical characteristics (body odor = 28%, inappropriate staring = 20%). In addition, among Japanese patients with OTKS, the highest percentage comprised those concerned about their facial expressions (45%) [20]. Although these results suggest that OTKS related to facial stiffness is relatively common, research has focused more on ORS and fear of eye contact.

The fourth is the fear of embarrassing others and making others uncomfortable through one’s deformed or defective appearance, or *shubo-kyofu* in the traditional Japanese diagnostic system [3,20,26,36]. This is classified as a subtype of TKS in Japan [29], and is closely associated with body dysmorphic disorder (BDD) [37,38]. However, while BDD includes the idea or delusion of reference, believing that others take special notice of or mock them because of their appearance [3], OTKS is focused on making others feel uncomfortable or offended by one’s physical appearance [16,20].

### Assessment and measurement of OTKS

The first TKS scale, developed by Kleinknecht et al. [18], has been widely used [39–41]. However, the items on this scale include symptoms of both offensive- and sensitive-type TKS, without a clear distinction between the two. Choy et al. [16] developed the TKS Questionnaire (TKSQ) comprising 30 items that assess 10 TKS symptoms, of which five were hypothesized to be specific to the offensive subtype (fear of stiff facial expression, body odor, inappropriate eye contact, intestinal gas, and physical appearance), and the other five common to both SAD and TKS (fears of blushing, body trembling, voice trembling, sweating, and making eye contact). TKSQ and its subscales were weakly to mildly correlated with psychopathological measurements of social anxiety and depression. Asakura et al. [19] developed the Social Anxiety/*Taijin-kyofu* Scale (SATS) that referenced the Yale-Brown Obsessive Compulsive Scale (Y-BOCS) to evaluate SAD symptoms, including “convinced TKS” (OTKS with a delusional component). Using a structured interview, SATS evaluates fear, avoidance behavior, and cognitive symptoms related to TKS and SAD on a 5-point scale ranging from 0 to 4. Application of SATS to 15 patients with TKS showed that the tool has high reliability based on Cronbach’s coefficient, inter-rater reliability, and test–retest reliability. SATS was also found to be correlated with the Clinical Global Impression severity assessment, confirming its validity.

Thus, various TKS scales have been developed. However, earlier scales assess general TKS symptoms, rather than specifically measuring OTKS [18]. Additionally, although TKSQ [16] effectively assesses each of the four OTKS subtypes, it does not assess the associated avoidance and safety behaviors [7–9]. Conversely, SATS [19] is a measure that can appraise the cognitive and behavioral symptoms associated with OTKS; however, it uses a structured interview method. Self-report scales have the advantage of providing participants with privacy in answering questions that they may find embarrassing to answer otherwise. Furthermore, none of the self-report scales include items that assess the “degree of confidence” in one’s thinking, although in severe cases of OTKS, thoughts can be delusional.

### Purpose of the study

This study aimed to develop and test the psychometric properties of a new self-report measure to assess OTKS (OTKS scale or OTKSS; Survey 1). It also intended to evaluate its test–retest reliability and correlation with the quality of life (Survey 2). The characteristics of OTKSS are as follows:

a) Assesses four types of OTKS (fear of body odor, inappropriate eye contact, inappropriate facial expressions, and defective physical appearance). As mentioned above, there are two types of TKS [19,20]: sensitive TKS and OTKS; the OTKSS aimed to assess OTKS only.
b) Assesses cognitive symptoms, including items on the frequency (e.g., *how frequently do you think that “my odor makes others feel uncomfortable?”*), confidence (e.g., *how much do you believe the thought “my odor makes others feel uncomfortable” to be true?*), and distress (e.g., *how distressed are you by the thought “my odor makes others feel uncomfortable?”*).
c) Assesses behavioral symptoms, including the frequency of avoidance and safety behavior use.

## Materials and methods

### Ethics statement

The studies (Survey 1 and Survey 2) reported in this paper were approved by the Ethics Committee of Taisho University (approval numbers: 22–23 and 23–12 for Survey 1 and 2, respectively). Online informed consent forms were signed by participants.

### Construction of the OTKSS

The scale was developed by a team of researchers from Japan and the United Kingdom specializing in TKS, SAD, obsessive-compulsive disorders (OCD), and psychosis. The OTKSS’s format was based on Y-BOCS [42–45], SATS [19], and Peters et al.’s Delusions Inventory (PDI) [46]. The 28 original items were formulated by the first six authors. To ensure that the OTKSS items reflected the symptoms of OTKS, we reviewed the literature on the definition and range of OTKS [16,19–21,26,28,37]. Consequently, four subscales were constructed representing the major symptoms. The subscales, in order, represent the concerns that others may be disturbed or offended by one’s body odor or intestinal gas (i.e., *jiko-shu-kyofu*); inappropriate eye contact (e.g., staring, sideways glances, shifting eyes; *jiko-shisen-kyofu*); inappropriate facial expression (e.g., stiff, drawn, sullen); and defective or deformed physical appearance.

Each of the four subscales contains seven items. The format of Items 1–3 was based on the PDI [46]. Item 1 (frequency) asked respondents how frequently they thought that a particular characteristic made others feel uncomfortable. Item 2 (conviction) asked respondents to rate the degree to which they believed their thoughts regarding the characteristic that made others uncomfortable. Item 3 (distress) asked respondents how distressed they were by their thoughts about the characteristic that they felt made others uncomfortable. Item 4 (avoidance) asked respondents how often they avoided social activities because of the concern of this characteristic offending others. Item 5 (safety behavior) asked respondents to select the safety behaviors they engaged in from multiple options. Item 6 (frequency of safety behavior) asked respondents how frequently they performed the safety behaviors selected in Item 5. Item 7 (interference in life from safety behavior) asked respondents how much the safety behaviors selected in Item 5 interfered with their social life. All seven items are asked for each of the four OTKS dimensions, giving the OTKSS 28 items in total, each of which (except Item 5, which involves selecting one’s own safety behavior) is rated on a four-point Likert scale. The total score for the remaining 24 items ranges from 0 to 96. A response of “Not at all” to Item 1 (Frequency) indicates that the respondent has not experienced that subtype of OTKS thinking, and the subsequent items in this dimension are automatically rated 0. Respondents were then asked to answer questions about other OTKS dimensions.

Finally, the face validity of the scale was adjusted and refined through discussions between the Japanese scale development team and UK researchers. After completing the original Japanese version, an English version of the scale was created, back-translated by a bilingual translator, and confirmed as having no major differences in meaning from the original Japanese version.

### Pre-testing the newly developed OTKSS

Once the list of items and instructions were agreed upon, the tool was tested among university students (*n *= 61, female = 34, male = 27, mean age = 21.082, SD = 3.393) who self-reported having experienced at least one form of OTKS. They provided feedback on the wording and comprehensibility of the items, relevance to their experiences, general acceptability of the measure, and feasibility of its completion. The feedback obtained was highly positive, with only minor amendments required to the wording. Based on the results of this pre-test and feedback from the participants, the authors confirmed that the text of the OTKS was written in a way that any university student could understand. In addition, Japanese Readability Test (an online tool in Japanese), which can provide an indication of a text’s reading level, was used to assess the grade level of the scale’s texts [47]. The results showed that our Japanese texts were understandable at a high-school level (12th grade). The participants in this pre-test did not participate in the subsequent surveys.

### Survey 1: Evaluation of the OTKSS’ psychometric properties

#### Participants.

The participants for this study met the following criteria: 1) Japanese University students aged 18–24 years, 2) determined as having SAD based on the LSAS-J cut-off values; and 3) reported one or more symptoms of OTKS. Based on Comrey and Lee’s [48] recommendations for adequate sample sizes in factor analysis (500 = very good; 1,000 or more = excellent), we attempted to recruit more than 500 participants who met the above criteria. Recruitment proceeded via flyers and announcements. The recruitment period for Survey 1 was from 30/09/2022–18/04/2023.

The study involved 811 university students in Japan (264 male, 530 female, 17 gender not reported; mean age = 19.55, SD = 1.270). Post-screening using the LSAS-J [49], 534 participants (137 male, 384 female, 13 gender not reported; mean age 19.58 years, SD = 1.307) were considered as having probable SAD based on the cut-off point in an earlier Japanese study (scores > 44) [49], and additional analyses were performed with this group.

#### Measurements.

a. **OTKSS** (created by the authors): the measure described above. In this study, we used the Japanese version of the TKSS.
b. **TKS scale** [18]: This self-report scale, traditionally used to measure general TKS, requires responses to 31 items on a 7-point Likert-type scale ranging from 1 (not at all true) to 7 (completely true). The total score for all items is calculated, with a higher score indicating a higher tendency toward TKS.
c. **LSAS Japanese version** (LSAS-J) [49,50]: This scale evaluates social anxiety symptoms (24 items, 4-point Likert-type scale). We used the self-report version, wherein the total score for all items is calculated, with higher scores indicating a greater tendency toward social anxiety.

#### Statistical analysis.

After developing the OTKSS, we applied the following analytical procedures to confirm its psychometric properties.

a) Factor structure and internal consistency were determined using exploratory factor analysis.
b) Correlation analyses were conducted for the OTKSS with social anxiety measured by LSAS-J [49] and the TKS scale [18].
c) Convergent and discriminant validity were examined by average variance extracted (AVE).

Statistical procedures were performed using IBM SPSS Version 27.0 (IBM Corp, 2020)

### Survey 2: Test–retest reliability of OTKSS and correlation with quality of life

#### Procedure and participants.

Survey 2 examined the test–retest reliability across a 4-week interval. The eligibility criteria for sample selection were: Japanese university students aged 18–24 years; met the criteria for SAD based on LSAS-J [49]; and reported one or more symptoms of OTKS. Recruitment was via flyers and announcements. The recruitment period for Survey 2 was from 10/11/2023–25/12/2023.

For T1, 242 Japanese students were recruited, of whom 144 (51 men, 90 women, 3 others; mean age = 19.18, SD = 0.98) met the eligibility criteria and completed the OTKSS at T1 and T2. At T2, in addition to OTKSS, the Japanese version of the Sheehan Disability Scale (SDS) [51,52], a short measure of disability and functional impairment, was also administered.

#### Statistical analysis.

Pearson’s correlation coefficient was used to describe the relationship between overall test scores at T1 and T2, for the total score and each individual OTKSS domain. Additionally, Pearson’s correlation coefficient was used to describe the relationship between overall OTKSS test scores at T2, and the SDS scores.

## Results

### Factor analysis of the OTKSS

An exploratory factor analysis was conducted for the OTKSS, wherein we checked the minimum average partial and eigenvalues of the squared multiple correlation. An initial factor analysis (promax rotation, maximum likelihood method) was performed, assuming four factors for the 24 items, excluding the four items on safety behavior (Items 5, 12, 19, and 26). The initial factor analysis produced five factors with eigenvalues greater than 1, with values of 9.738, 2.555, 2.313, 1.778, and 1.144, suggesting a five-factor solution using Kaiser’s [53] greater-than-one rule. Scree plot inflections suggested four- or five-factor models [54]. Additionally, parallel analysis [55] was conducted to compare whether the extracted eigenvalues were larger than the mean of those obtained from random generation [56]. The parallel analysis suggested retaining up to four factors. Taking the eigenvalues, scree plots, and parallel analysis into account, four- and five-factor models were tested. Theoretical models of OTKS and factor interpretability were considered while deciding how many factors to retain [57].

#### Four-factor model.

The factor loadings for all the items were greater than.50 (Table 1). Additionally, the cumulative proportion of variance explained by all four factors was 68.27%. Factor 1 consisted of all items related to eye contact, Factor 2 consisted of all items related to appearance, Factor 3 consisted of all items related to facial expressions, and Factor 4 consisted of all items related to body odor. Cronbach’s α values for Factors 1–4 were.905,.923,.899, and.872, respectively; the McDonald’s ω values were.912,.929,.909, and.882, respectively. Based on these results, it was determined that the four-factor structure was appropriate.

**Table 1:** Exploratory factor analysis for OTKSS (n = 534).

No.	Items	F1	F2	F3	F4	Comm.
**Factor 1 Eye contact (α = .872, **ω** = .879)**						
8	How frequently have you thought, “My eye contact (e.g., staring at others, glances, sideways glances, shifting eyes) makes others feel uncomfortable?”	**.894**	−.109	−.007	−.032	.684
13	Based on your response to the above question, overall, how often have you done these over the past week?	**.831**	.015	−.004	−.079	.649
10	How distressed are you by the thought “My eye contact makes others feel uncomfortable?”	**.816**	−.023	.038	−.004	.681
9	How much do you believe the thought “My eye contact makes others feel uncomfortable” to be true?	**.782**	−.028	.001	.098	.662
14	Based on your response to question 12, how much has doing these behaviors interfered with your social life (working, going to school, social interaction, etc.) over the past week?	**.690**	.158	−.022	−.004	.591
11	How often have you avoided going out, interacting with others, or engaging in other activities because of concerns about your eye contact?	**.670**	.007	.029	.063	.519
**Factor 2 Appearance (α = .905, **ω** = .910)**						
22	How frequently have you thought, “My defects or flaws in appearance make others feel uncomfortable?”	−.073	**.914**	.005	.021	.790
24	How distressed are you by the thought “My defects or flaws in appearance make others feel uncomfortable?”	−.086	**.909**	.025	.002	.776
23	How much do you believe the thought “My defects or flaws in appearance make others feel uncomfortable” to be true?	−.070	**.852**	.024	.042	.717
27	Based on your responses to the above question, overall, how often have you done these over the past week?	.028	**.806**	−.029	−.085	.601
25	Over the past week, how often have you avoided going out, interacting with others, or engaging in other activities because of your concerns about your appearance?	.157	**.731**	−.057	.016	.638
28	Based on your response to Question 26, how much has doing these behaviors interfered with your social life (working, going to school, social interaction, etc.) over the past week?	.057	**.723**	.046	−.014	.600
**Factor 3 Facial expression (α = .899, **ω** = .908)**						
15	How frequently have you thought, “My facial expressions (e.g., tense or stiff, twitchy, sullen) make others feel uncomfortable?”	−.017	−.091	**.949**	.009	.808
17	How distressed are you by the thought “My facial expressions make others feel uncomfortable?”	−.017	−.011	**.846**	.020	.704
16	How much do you believe the thought “My facial expressions make others feel uncomfortable” to be true?	−.074	.016	**.835**	−.004	.640
20	Based on your response to the above question, overall, how often have you done these over the past week?	.031	−.004	**.743**	.009	.584
21	Based on your response to Question 19, how much has doing these behaviors interfered with your social life (working, going to school, social interaction, etc.) over the past week?	.140	.145	**.552**	−.045	.512
18	How often have you avoided going out, interacting with others, or engaging in other activities because of your concerns about your facial expressions?	.123	.098	**.528**	.006	.450
**Factor 4 Body odor (α = .923, **ω** = .928)**						
1	How frequently have you thought, “My odor makes others feel uncomfortable”?	−.050	−.030	−.025	**.895**	.730
2	How much do you believe the thought “My odor makes others feel uncomfortable” to be true?	−.028	−.033	.040	**.808**	.642
3	How distressed are you by the thought “My odor makes others feel uncomfortable?”	.003	−.071	−.020	**.783**	.562
6	Based on your response to the above question, overall, how often have you done these over the past week?	.019	−.067	.043	**.665**	.446
4	How often have you avoided going out, interacting with others, or engaging in other activities because of your concerns about body odor?	.026	.142	−.026	**.611**	.464
7	Based on your response to question 5, how much has doing these behaviors interfered with your social life (working, going to school, social interaction, etc.) over the past week?	.086	.152	−.002	**.564**	.470
	Proportion of variance explained	40.57	10.55	9.64	7.41	68.27

#### Five-factor model.

The factor loadings for all the items were greater than.50. Additionally, the cumulative proportion of variance explained by all four factors was 73.04%. However, the factors were not clearly interpretable because Factor 1 consisted of the item related to appearance, and Factor 5 consisted of the item related to body odor, whereas Factors 3, 4, and 5 consisted of a mix of items related to facial expressions and eye contact.

#### Final model selection.

Based on the factor analysis, existing theories [19,20], and previous psychological measurements about OTKS [16,19], the four-factor model was chosen as the most parsimonious solution.

### Correlation analysis

The correlation analysis between the OTKSS and the other social anxiety measures (Table 2) showed a significant moderate to high correlation with the TKS Scale (*r* = .737) and LSAS-J (*r* = .482). Moreover, the four subscales of the OTKSS were also significantly moderately correlated with the TKS Scale (*r *= .458–.613, *p* < .001), and low to moderately correlated with LSAS-J (*r* = 286–.415, *p* < .001).

**Table 2:** Correlation table with square root of AVE on diagonal (n = 534).

	CR	AVE	OTKSS total	Body odor	Eye contact	Facial expression	Appearance
Body odor	.870	.533	.688^**^	**.730**			
Eye contact	.905	.616	.788^**^	.386^**^	**.785**		
Facial expression	.886	.575	.797^**^	.406^**^	.561^**^	**.758**	
Appearance	.927	.682	.786^**^	.367^**^	.471^**^	.498^**^	**.826**
TKS Scale			.737^**^	.458^**^	.605^**^	.572^**^	.613^**^
LSAS-J			.482^**^	.286^**^	.415^**^	.361^**^	.406^**^
Mean			27.017	7.670	6.200	6.069	7.077
SD			16.588	5.024	5.377	5.136	6.104

OTKSS = Offensive Taijin-Kyofu-Sho scale; TKS Scale = Taijin-Kyofu-Sho Scale; LSAS-J = Liebowitz Social Anxiety Scale, Japanese version; SD = Standard Deviation; CR = Composite Reliability; AVE = Average Variance Extracted; Values in bold are the square root of AVE.

**p < .001

### Reliability and validity

According to Hair et al. [58], the acceptable composite reliability (CR) is > 0.7. The CR values of the four subscales of the OTKSS ranged from.870 to.927, indicating good reliability. Additionally, the AVE for all factors met the minimum requirement for convergent validity, which is 0.5 [59]. Finally, the square root of AVE for the scales was greater than the other inter-factor correlations, establishing discriminant validity of the constructs [60].

### Test–retest reliability of the OTKSS

There was a high positive correlation between T1 and T2 OTKSS test scores (*r* [142] =.771, *p* < .001), and OTKSS test scores for the subscales of body odor (*r* [142] =.725, *p* < 0.001) and physical appearance (*r* = [142] = 0.767, *p *< 0.001). There was a moderate positive correlation between T1 and T2 OTKSS scores for the subscales of eye contact (*r* [df] =.693, *p* < 0.001) and physical appearance (*r* = [142] = 0.690, p < 0.001).

A post hoc power analysis was conducted with IBM SPSS Version 27.0 for the final sample size (*n* = 144) and Pearson’s correlation coefficient with α = 0.05. We used the yielded effect size from the correlation coefficient (*r* = .771) between T1 and T2 of the OTKSS total score. Statistical power was 1.00 (1 − β > 0.95), suggesting that our sample size was adequate.

### Correlation between the OTKSS, and disability and functional impairment

The correlation coefficient between OTKSS and the total SDS score was *r* = .707; correlations of SDS with the body odor subscale was *r* = .566 (*p* < .001), eye contact was *r *= .636 (*p* < .001), facial expression was *r* = .572 (p < .001), and appearance was *r* = .643 (*p* < .001).

## Discussion

This study aimed to develop a new self-report measure for assessing OTKS using a sample of Japanese students who have probable SAD. Factor structure and internal consistency were determined using exploratory factor analysis, correlation analyses were conducted for the OTKSS with social anxiety measured by LSAS-J [49] and the TKS scale [18], and convergent and discriminant validity were examined by AVE.

Based on the exploratory factor analysis, the four-factor structure of the OTKSS was deemed appropriate. OTKSS scores were positively correlated with those of the LSAS, the TKS Scale, and the SDS. In terms of reliability, Cronbach’s α and CR were acceptable, indicating good internal consistency. In addition, the OTKSS demonstrated acceptable test–retest reliability. In terms of convergent validity, all of the reported AVE values for the OTKSS subscales were within the acceptable range. Furthermore, since the square root of AVE for the scales was greater than that of the other inter-factor correlations, the discriminant validity of the OTKSS was established. These results confirm the psychometric properties of the OTKSS, indicating that the scale has good validity and reliability for the Japanese students’ sample. Therefore, the OTKSS has the potential to contribute significantly to psychopathological research and psychological treatments related to OTKS. However, these findings provide preliminary evidence because we used a sample limited sample of Japanese university students. Further structural model verification using a different data set is required for factor analysis.

The OTKSS has several novel advantages. First, it can assess the four typical types of OTKS. By dividing TKS symptoms into dimensions, it is possible to elicit symptom types that are inherently ambiguous, and thereby assess multiple coexisting TKS symptoms in clinical settings. Although the comorbidity rates of OTKS remain unclear, studies show that patients with SAD complain of multiple OTKS symptoms [16,20]. Therefore, the OTKSS is a comprehensive assessment tool for OTKS that is useful for understanding the characteristics of its symptoms.

Second, the OTKSS can assess not only cognitive but also behavioral symptoms of OTKS, such as safety and avoidance. Using this measure, the type of safety behavior can be specified, and its manifestation evaluated in clinical settings. In the future, we intend to use this scale to clarify the types of safety behaviors that are particularly associated with severe OTKS symptoms in a longitudinal study.

Third, the OTKSS measures the frequency, conviction, and distress related to cognitive symptoms. Notably, OTKS can include delusions, wherein patients strongly believe that they are causing harm to others. Assessing the conviction of thought is important as it relates to symptom severity.

Fourth, the OTKSS comprises a broad range of items that can measure OTKS tendencies among university students. OTKS has been identified in 1–6% of university students and community populations who do not have clinical diagnoses [24,25,34], and, similar to other anxiety and depressive disorders, there is a continuum between healthy subjects and clinical groups. Therefore, OTKSS has the advantage of being able to assess clinical groups and the general population and may be useful in cross-sectional surveys and studies of analogue samples.

Finally, the scale was developed by a team of researchers specializing in TKS, SAD, OCD, and psychosis. Therefore, the content validity of the scale is adequately established. In addition, the scale development team involved not only Japanese but also British researchers, resulting in a tool that can be used internationally.

### Limitations and future research

First, the sample in this study was a “caseness” SAD group identified by the LSAS self-report measure and did not necessarily have clinical diagnoses of SAD. Future studies could employ a two-phase survey, whereby the first comprises screening for SAD using self-report measures (e.g., LSAS), and the second involves follow-up with standardized diagnostic procedures.

Second, the participants for this study were limited to Japanese university students with a highly restricted age range (18–24 years). Given that the psychometric work of this paper was conducted only with this population, our findings related to the preliminary evidence of reliability and validity of the OTKSS apply to this population only. Although this study indicated that the four-factor structure of the OTKSS was appropriate, exploratory factor analysis was conducted using only a university student sample. Therefore, the preliminary structural validity was established only for this population. Future research should examine the validity of the four-factor model indicated in this study through confirmatory factor analysis using an independent dataset. Third, we did not assess discriminant validity in relation to other anxiety disorders, other psychiatric disorders, and healthy subjects. These must be examined in separate future studies.

Fourth, this study aimed to develop a scale and not compare TKS symptoms among ethnicities. Therefore, we did not obtain detailed racial data on Japanese university students. As the manifestation of TKS may vary according to race, future research is needed to clarify these differences. British researchers participated in the working group that developed the OTKSS, and the English version of the scale was also completed. A future comparative study has been planned between Japan and the United Kingdom.

Finally, OTKS is thought to be related not only to SAD but also to OCD and delusional thoughts. However, to avoid burdening the participants with excessive questionnaires, we only examined the relationship between OTKS and SAD. Future research should examine the relationships among OTKS, OCD, and delusional thoughts.

## Conclusions

This study developed a self-report measure for assessing OTKS (the OTKSS). The findings from Survey 1 and 2 indicate that the OTKSS is a reliable and valid instrument for assessing OTKS. In addition, analysis of the data obtained from the scale’s administration suggests the harmful effects of avoidance and safety behavior use. However, a key limitation of this study is that the sample consisted solely of Japanese university students. As the psychometric analyses were conducted exclusively on this population, the preliminary evidence of reliability and validity is applicable only within this context. Future research should assess the validity of these measures using larger and more diverse samples, including clinical populations. An English version of the scale was simultaneously prepared and will be tested.

## Supporting information

S1 Appendix Offensive Taijin-Kyofu-Sho Scale (OTKSS). (DOCX)
