# Supplementary material for: The development and validation of the Offensive-Type Taijin-Kyofu-Sho Scale (OTKSS): A preliminary study using a sample of Japanese University Students
Source: PLOS Ment Health. 2025 May 28;2(5):e0000223. doi: 10.1371/journal.pmen.0000223 (PMC12798263; doi:10.1371/journal.pmen.0000223)
Supplement: S1 Appendix — (DOCX) [file pmen.0000223.s001.docx]

**OFFENSIVE TAIJIN-KYOFU-SHO SCALE (OTKSS), English version.**

**Concerns about your odors**

The following questions relate to your concerns about personal odors over the past week (including today). Some examples are body odor, bad breath, sweat odor, underarm odor, foot odor, clothing odor, odor from the lower half of your body, flatulence, or any other concerns about bodily odor. For each question, please choose the response that best describes you.

1. Over the past week, how frequently have you thought, “My odor (e.g., body odor, bad breath, sweat odor, underarm odor, foot odor, clothing odor, odor from the lower half of the body, flatulence, etc.) makes others feel uncomfortable”?
2. Never *(If respondents check “Never,” the answers to questions 2 to 7 will be automatically entered as 0*)
3. Occasionally
4. Sometimes
5. Often
6. Very often
7. How much do you believe the thought “My odor makes others feel uncomfortable” to be true?
   - - 1. I do not believe it is true (the probability that my thought is true is almost 0%)
       2. I believe it a little bit (approximately 10%–30%)
       3. Half in doubt (approximately 40%–60%)
       4. I mostly believe it to be true (approximately 70%–90%)
       5. I absolutely believe my odor makes others feel uncomfortable (almost 100%)
8. How distressed are you by the thought “My odor makes others feel uncomfortable?”
   - 1. Not at all
     2. A little
     3. To some extent
     4. Quite a bit
     5. Very much
9. Over the past week, how often have you avoided going out, interacting with others, or engaging in other activities because of your concerns about body odor?
   - - - 1. Never (the probability of avoiding is almost 0%)
     1. Occasionally avoided (approximately 10%–30%)
     2. Sometimes avoided (approximately 40%–60%)
     3. Often avoided (approximately 70%–90%)
     4. Avoided almost all the time (almost 100%)
10. Over the past week, have you done any of the behaviors listed below because of concerns about your odor? Please check all that apply (Multiple answers allowed).
11. Hiding the odor with deodorants, perfumes, lotions, powders, mouthwash, mints, gum, creams, ointments, medicines, etc.
12. Sniffing the concerned area of my body and its surrounding areas to check for odor
13. Paying attention to others’ gaze or reactions (e.g., checking if others are turning away from me, sniffing, coughing, avoiding me, talking quietly about me, or responding negatively)
14. Showering carefully to eliminate the odor or thoroughly washing clothes
15. Washing specific parts of the body or brushing teeth meticulously
16. Changing clothes to hide the odor or choosing clothes that prevent or conceal sweating
17. Airing out the concerned area to eliminate the odor or staying near a window
18. Staying as far away from other people as possible
19. Wearing a mask to hide bad breath
20. Avoiding talking to hide bad breath
21. Keeping my arms fixed by the side of the body or avoiding raising the arms
22. Apologizing or making excuses (e.g., “It’s hot in here isn’t it?” “Sorry, I’ve been exercising,” “Is there an odor coming from over there?”)
23. Following a special diet or taking supplements to try and change body odor/avoiding or limiting foods and drinks that might increase body odor
24. Searching for information on how to alleviate body odor (e.g., reading books and articles, browsing the Internet)
25. Other (Please describe)
26. Nothing/does not apply (*If respondents select “Nothing,” the answers to questions 6 to 7 will be automatically entered as 0*)
27. Based on your response to the above question, overall, how often have you done these over the past week?
    - - - 1. Almost never (If you answer “Nothing/does not apply” in the above list, the score here will be automatically entered as “0”.)
          2. Occasionally
          3. Sometimes
          4. Often
          5. Very often
28. Based on your response to question 5, how much has doing these behaviors interfered with your social life (working, going to school, social interaction, etc.) over the past week?
    - 1. Not at all
      2. A little
      3. To some extent
      4. Quite a bit
      5. Very much

**Concerns about eye contact**

The following questions relate to your concerns about eye contact over the past week (including today). Some examples include staring at others, glances, sideways glances, and shifting eyes during conversations.

1. Over the past week, how frequently have you thought, “My eye contact (e.g., staring at others, glances, sideways glances, shifting eyes) makes others feel uncomfortable?”
2. Never (*If respondents select “Never,” the answers to questions 9 to 14 will be automatically entered as 0*)
3. Occasionally
4. Sometimes
5. Often
6. Very often
7. How much do you believe the thought “My eye contact makes others feel uncomfortable” to be true?
8. I do not believe it is true (the probability that my thought is true is almost 0%)
9. I believe it a little bit (approximately 10%–30%)
10. Half in doubt (approximately 40%–60%)
11. I mostly believe it to be true (approximately 70%–90%)
12. I absolutely believe my eye contact makes others feel uncomfortable (almost 100%)
13. How distressed are you by the thought “My eye contact makes others feel uncomfortable?”
14. Not at all
15. A little
16. To some extent
17. Quite a bit
18. Very much
19. Over the past week, how often have you avoided going out, interacting with others, or engaging in other activities because of concerns about your eye contact?
20. Never (the probability of avoiding is almost 0%)
21. Occasionally avoided (approximately 10%–30%)
22. Sometimes avoided (approximately 40%–60%)
23. Often avoided (approximately 70%–90%)
24. Avoided almost all the time (almost 100%)
25. Over the past week, have you done any of the behaviors listed below because of concerns about your eye contact? Please check all that apply (Multiple answers allowed).
26. Covering my face with something (hand, hair, glasses, a mask, etc.)
27. Avoiding eye contact
28. Hiding my glance by looking down or closing my eyes, etc.
29. Paying attention to others' gaze or reactions (e.g., checking to see if others are turning away from me, avoiding me, talking quietly about me, or responding negatively)
30. Avoiding facing other people
31. Asking a trusted person (family or friend) to make sure my eye contact is not strange
32. Positioning myself so as not to be noticed (standing at the back/in the corner)
33. Trying to picture how I appear to others
34. Collecting information on how to improve my eye contact (reading books and articles, browsing the Internet, etc.)
35. Other (Please describe)
36. Nothing/does not apply (*If respondents check “Nothing,” the answers to questions 13 to 14 will be automatically entered as 0*)
37. Based on your response to the above question, overall, how often have you done these over the past week?
    - - 1. Almost never (If respondents answer “Nothing/does not apply” in the above list, the score here will be automatically entered as 0)
        2. Occasionally
        3. Sometimes
        4. Often
        5. Very often
38. Based on your response to question 12, how much has doing these behaviors interfered with your social life (working, going to school, social interaction, etc.) over the past week?
39. Not at all
40. A little
41. To some extent
42. Quite a bit
43. Very much

**Concerns about your** **facial expressions**

The following questions relate to your concerns about your facial expressions over the past week (including today). Some examples are a tense, stiff, twitchy, or sullen expression, or any other concerns about your facial expressions.

1. Over the past week, how frequently have you thought, "My facial expressions (e.g., tense or stiff, twitchy, sullen) make others feel uncomfortable?”
2. Never (*If respondents select “Never,” the answers to questions 16 to 21 will be automatically entered as 0*)
3. Occasionally
4. Sometimes
5. Often
6. Very often
7. How much do you believe the thought "My facial expressions make others feel uncomfortable" to be true?
8. I do not believe it is true (the probability that my thought is true is almost 0%)
9. I believe it a little bit (approximately 10%–30%)
10. Half in doubt (approximately 40%–60%)
11. I mostly believe it to be true (approximately 70%–90%)
12. I absolutely believe my facial expression makes others feel uncomfortable (almost 100%)
13. How distressed are you by the thought "My facial expressions make others feel uncomfortable?”
14. Not at all
15. A little
16. To some extent
17. Quite a bit
18. Very much
19. Over the past week, how often have you avoided going out, interacting with others, or engaging in other activities because of your concerns about your facial expressions?
20. Never (the probability of avoiding is almost 0%)
21. Occasionally avoided (approximately 10%–30%)
22. Sometimes avoided (approximately 40%–60%)
23. Often avoided (approximately 70%–90%)
24. Avoided almost all time (almost 100%)
25. Over the past week, have you done any of the behaviors listed below because of concerns about your facial expressions? Please check all that apply (Multiple answers allowed).
26. Covering my face with something (hand, hair, glasses, mask, etc.)
27. Avoiding face-to-face contact with other people
28. Trying to change my expressions
29. Turning my head away from others
30. Paying attention to others’ gaze or reactions (e.g., checking if others were turning away from me, avoiding me, talking quietly about me, or responding negatively)
31. Positioning myself so as not to be noticed (standing at the back/in the corner)
32. Trying to picture how I appear to others
33. Asking a trusted person (family or friend) to ensure my facial expressions are not strange
34. Buying cosmetics or accessories to hide my facial expressions
35. Collecting information on how to improve my facial expressions (reading books and articles, browsing the Internet, etc.)
36. Other (Please describe)
37. Nothing/does not apply (*If respondents check “Nothing,” the answers to questions 20 to 21 will be automatically entered as 0*)
38. Based on your response to the above question, overall, how often have you done these over the past week?
39. Almost never (If respondents answer “Nothing/does not apply” in the above list, the score here will be automatically entered as 0)
40. Occasionally
41. Sometimes
42. Often
43. Very often
44. Based on your response to Question 19, how much has doing these behaviors interfered with your social life (working, going to school, social interaction, etc.) over the past week?
45. Not at all
46. A little
47. To some extent
48. Quite a bit
49. Very much

**Concerns about defects or flaws in your appearance**

The following questions relate to concerns about defects or flaws in your appearance over the past week (including today). Examples include areas of your face or body that you consider *ugly, unattractive,* or *inferior*.

1. Over the past week, how frequently have you thought, "My defects or flaws in appearance make others feel uncomfortable?”
2. Never (*If respondents select “Never,” the answers to questions 23 to 28 will be automatically entered as 0*)
3. Occasionally
4. Sometimes
5. Often
6. Very often
7. How much do you believe the thought “My defects or flaws in appearance make others feel uncomfortable" to be true?
8. I do not believe it is true (the probability that my thought is true is almost 0%)
9. I believe it a little bit (approximately 10%–30%)
10. Half in doubt (approximately 40%–60%)
11. I mostly believe it to be true (approximately 70%–90%)
12. I absolutely believe my appearance makes others feel uncomfortable (almost 100%)
13. How distressed are you by the thought "My defects or flaws in appearance make others feel uncomfortable?”
14. Not at all
15. A little
16. To some extent
17. Quite a bit
18. Very much
19. Over the past week, how often have you avoided going out, interacting with others, or engaging in other activities because of your concerns about your appearance?
20. Never (the probability of avoiding is almost 0%)
21. Occasionally avoided (approximately 10%–30%)
22. Sometimes avoided (approximately 40%–60%)
23. Often avoided (approximately 70%–90%)
24. Avoided almost all time (almost 100%)
25. Over the past week, have you done any of the behaviors listed below because of concerns about your appearance? Please check all that apply (Multiple answers allowed).
26. Checking my appearance in the mirror
27. Grooming myself carefully (e.g., repeatedly applying makeup, styling or combing hair, plucking hair, or shaving)
28. Scrutinizing and comparing others' appearances with my own.
29. Hiding visible body parts of concern (covering with clothing, hats, tanning, makeup, hair, beards, hands, masks, etc.)
30. Touching or measuring the body parts I am concerned about (contour of the face, perceived acne, scars, wrinkles, legs, stomach, body fat, etc.)
31. Paying attention to others’ gaze or reactions (e.g., checking if others were turning away from me, avoiding me, talking quietly about me, or responding negatively)
32. Trying to picture how I appeared to others
33. Asking a trusted person (family or friend) to ensure my appearance is not strange
34. Engaging in unusual food intake or excessive dieting to improve my appearance
35. Exercising to improve my appearance
36. Compulsively shopping for beauty products
37. Undergoing or planning surgical enhancements (cosmetic surgery, dental procedures)
38. Collecting information on improving my appearance (reading books and articles, browsing the Internet, etc.)
39. Positioning myself so as not to be noticed (standing at the back or in the corner of a room)
40. Other (Please describe)
41. Nothing/does not apply (*If respondents check “Nothing,” the answers to questions 27 to 28 will be automatically entered as 0*)
42. Based on your responses to the above question, overall, how often have you done these over the past week?
43. Almost never (If respondents answer “Nothing/does not apply” in the above list, the score here will be automatically entered as 0)
44. Occasionally
45. Sometimes
46. Often
47. Very often
48. Based on your response to Question 26, how much has doing these behaviors interfered with your social life (working, going to school, social interaction, etc.) over the past week?
49. Not at all
50. A little
51. To some extent
52. Quite a bit
53. Very much

**Scoring**: Four subscale scores and one total score can be calculated.

— Body Odor: Sum of the scores for items 1, 2, 3, 4, 6, and 7.

— Eye Contact: Sum of the scores for items 8, 9, 10, 11, 13, and 14.

— Facial Expression: Sum of the scores for items 15, 16, 17, 18, 20, and 21.

— Appearance: Sum of the scores for items 22, 23, 24, 25, 27, and 28.

― Total score: Sum of the four subscales.

**OFFENSIVE TAIJIN-KYOFU-SHO SCALE (TKSS), Japanese version**

自分からでる臭いに関する心配について

以下の質問では、ここ1週間程度（本日を含む）における、あなたの臭いに関する心配について伺います。臭いの例としては、体臭、口臭、汗の臭い、脇、足、衣類、下半身からでる臭い、おなら等があります。これらの例以外にも、自分からでる臭いであれば全て該当します。以下の質問をよく読んで、当てはまる回答を数字で選んでお答えください。

1. ここ1週間、「自分の臭い（例： 体臭、口臭、汗の臭い、脇、足、衣類、下半身からでる臭い、おなら等）が他の人に嫌な感じを与えている」という心配について、どのくらいの頻度で考えましたか？
2. 全く考えなかった *ここで0と回答した場合、2－7の回答は全て0点となり、8の質問（視線）へスキップする
3. すこし考えた
4. ときどき考えた
5. たびたび考えた
6. とても頻繁に考えた
7. 「自分の臭いが他の人に嫌な感じを与えている」という考えが事実である（実際に嫌な感じを与えている）と、どのくらい思いますか？
8. 事実ではないと思う（その考えが事実である可能性はほぼ0％）
9. すこしは事実だと思う（10～30％程度）
10. 半信半疑（40～60％程度）
11. おおよそ事実だと思う（70～90％程度）
12. 私の臭いは実際に他人に嫌な感じを与えていると確信する（ほぼ100％）
13. 「自分の臭いが他の人に嫌な感じを与えている」という考えによって、どのくらい、あなたは苦痛を感じますか？
    - 1. 全くない
      2. すこし苦痛
      3. ある程度苦痛
      4. けっこう苦痛
      5. とても苦痛
14. ここ1週間、自分の臭いに関する心配が原因で、何かをしたり、どこかに出かけたり、人と関わる状況を避けたりすることがありましたか？
15. 全くない（避ける確率はほぼ0％）
16. すこし避けた（10－30％程度）
17. ときどき避けた（40－60％程度）
18. たびたび避けた（70－90％程度）
19. ほとんど全てのことを避けた（ほぼ100％避ける）
20. ここ1週間、自分の臭いに関する心配が原因で、以下のリストにある行動をしましたか？実際に行ったことがあるものを「すべて」選んでください（複数回答あり）。
    1. 臭いを隠すためのアイテムを使用する（消臭剤、香水、ローション、パウダー、マウスウォッシュ、ミント、ガム、クリーム、塗り薬、専用の医薬品、等）
    2. 臭いが気になる体の部位やその周辺の匂いを嗅ぐ
    3. 他人の目を気にする / 他人の反応を確認する（他人が顔をそむけたり、鼻をすすったり、咳払いをしたり、自分を避けたり、自分について小声で話していたり、不快そうな態度をしていないか等を確認する）
    4. 臭いを消すために入念にシャワーを浴びる / 着ていた服等を入念に洗濯する
    5. 身体の特定の部位を集中的に洗う / 入念に歯磨きをする
    6. 臭いを隠すために服を着替える / 臭いを防いだり隠したりできる服を選ぶ
    7. 臭いを消すために部屋の換気をする / 窓際にいる
    8. 他人にできるだけ近寄らない
    9. （口臭を隠すために）マスクをする
    10. あまりしゃべらないようにする
    11. 脇を締めて、腕を上げないようにする
    12. 臭いについて謝る / 言い訳をする（例：「ここ暑すぎじゃない？」、「すみません、運動してきたばかりで」、「向こうから変な臭いがしない？」等）
    13. 体臭を改善するための特別な食事やサプリメントをとる / 体臭が強くなりそうな飲食物を避ける
    14. 体臭を改善するための情報を集める（本や記事を読む / ネット等で調べる）
    15. その他（　　　　　　　　　　　　　　　　　　　　　　　　）
    16. 特に何もしない *この回答を選択した場合、6－7の回答は全て0点となり、8の質問（視線）へスキップ
21. 上記のリストで選択した行動を、ここ1週間、どのくらいの頻度で行いましたか？（上記にて複数の行動を選択した場合は、総合的にみて、それらの行動をどのくらい行うのかを答えてください）
    1. ほとんど行わなかった
    2. すこし行った
    3. ときどき行った
    4. たびたび行った
    5. とても頻繁に行った
22. 上記のリストで選択した行動は、ここ1週間、どのくらい社会生活（例： 仕事、学業、他人との交流など）の妨げになりましたか？（複数の行動を選択した場合は、総合的にみて、それらの行動がどのくらい妨げになっているのかを答えてください）
23. 全く妨げになっていなかった
24. すこし妨げになった
25. ある程度の妨げになった
26. けっこう妨げになった
27. かなり妨げになった

自分の視線に関する心配について

以下の質問では、ここ1週間程度における、あなたの視線に関する心配について伺います。視線の例としては、他人と目を合わせたり、他人をチラリと見たり、横目で見たり、会話中に目が泳いだりする、等があります。これらの例以外にも、自分の視線に関するものは、全て該当します。

1. ここ1週間、「自分の視線（例： 他人と目を合わせる、他人をチラリと見る, 横目で見る、会話中に目が泳ぐ、等）が他の人に嫌な感じを与えている」という心配について、どのくらいの頻度で考えましたか？
2. 全く考えなかった *ここで0と回答した場合、 9－14の回答は全て0点となり、15の質問（表情）へスキップする
3. すこし考えた
4. ときどき考えた
5. たびたび考えた
6. とても頻繁に考えた
7. 「自分の視線が他の人に嫌な感じを与えている」という考えが事実である（実際に嫌な感じを与えている）と、どのくらい思いますか？
8. 事実ではないと思う（その考えが事実である可能性はほぼ0％）
9. すこしは事実だと思う（10～30％程度）
10. 半信半疑（40～60％程度）
11. おおよそ事実だと思う（70～90％程度）
12. 私の臭いは実際に他人に嫌な感じを与えていると確信する（ほぼ100％）
13. 「自分の視線が他の人に嫌な感じを与えている」という考えによって、どのくらい、あなたは苦痛を感じますか？
14. 全くない
15. すこし苦痛
16. ある程度苦痛
17. けっこう苦痛
18. とても苦痛
19. ここ1週間、自分の視線に関する心配が原因で、何かをしたり、どこかに出かけたり、人と関わる状況を避けたりすることがありましたか？
20. 全くない（避ける確率はほぼ0％）
21. すこし避けた（10－30％程度）
22. ときどき避けた（40－60％程度）
23. たびたび避けた（70－90％程度）
24. ほとんど全てのことを避けた（ほぼ100％避ける）
25. ここ1週間、自分の視線に関する心配が原因で、以下のリストにある行動をしましたか？実際に行ったことがあるものを「すべて」選んでください（複数回答あり）
    1. 何かで顔を隠す（自分の手や髪、メガネ、マスクなどを使って隠す）
    2. 他人と目を合わせないようにする
    3. 下を向いたり、目をつぶったりして視線を隠す
    4. 他人の目を気にする / 他人の反応を確認する（他人が顔をそむけたり、自分を避けたり、自分について小声で話していたり、不快そうな態度をしていないか等を確認する）
    5. 他人と向かい合わせにならないようにする
    6. 信頼できる人（家族や友人）に自分の視線が変ではないか質問をして確認する
    7. 目立たない位置にいるようにする（後ろの方や部屋の隅にいる）
    8. 自分が他人からどう見られているのかを想像する
    9. 視線を改善するための情報を集める（本や記事を読む / ネット等で調べる）
    10. その他（　　　　　　　　　　　　　　　　　　　　　　　　）
    11. 特に何もしない *この回答を選択した場合、13－14の回答は全て0点となり、15の質問（表情）へスキップする
26. 上記のリストで選択した行動を、ここ1週間、どのくらいの頻度で行いましたか？（上記にて複数の行動を選択した場合は、総合的にみて、それらの行動をどのくらい行うのかを答えてください）
27. ほとんど行わなかった
28. すこし行った
29. ときどき行った
30. たびたび行った
31. とても頻繁に行った
32. 上記のリストで選択した行動は、ここ1週間、どのくらい社会生活（例： 仕事、学業、他人との交流など）の妨げになりましたか？（複数の行動を選択した場合は、総合的にみて、それらの行動がどのくらい妨げになっているのかを答えてください）
33. 全く妨げになっていなかった
34. すこし妨げになった
35. ある程度の妨げになった
36. けっこう妨げになった
37. かなり妨げになった

自分の表情に関する心配について

以下の質問では、ここ1週間程度における、あなたの表情に関する心配について伺います。表情の例としては、緊張してこわばった表情、引きつった表情、不機嫌そうな表情などがあります。これらの例以外にも、自分の表情に関するものは、全て該当します。

1. ここ1週間、「自分の表情（例： 緊張してこわばった表情、引きつった表情、不機嫌そうな表情など）が他の人に嫌な感じを与えている」という心配について、どのくらいの頻度で考えましたか？
2. 全く考えなかった *ここで0と回答した場合、16－21の回答は全て0点となり、22の質問（容姿）へスキップする
3. すこし考えた
4. ときどき考えた
5. たびたび考えた
6. とても頻繁に考えた
7. 「自分の表情が他の人に嫌な感じを与えている」という考えが事実である（実際に嫌な感じを与えている）と、どのくらい思いますか？
8. 事実ではないと思う（その考えが事実である可能性はほぼ0％）
9. すこしは事実だと思う（10～30％程度）
10. 半信半疑（40～60％程度）
11. おおよそ事実だと思う（70～90％程度）
12. 私の臭いは実際に他人に嫌な感じを与えていると確信する（ほぼ100％）
13. 「自分の表情が他の人に嫌な感じを与えている」という考えによって、どのくらい、あなたは苦痛を感じますか？
14. 全くない
15. すこし苦痛
16. ある程度苦痛
17. けっこう苦痛
18. とても苦痛
19. ここ1週間、自分の表情に関する心配が原因で、何かをしたり、どこかに出かけたり、人と関わる状況を避けたりすることがありましたか？
20. 全くない（避ける確率はほぼ0％）
21. すこし避けた（10－30％程度）
22. ときどき避けた（40－60％程度）
23. たびたび避けた（70－90％程度）
24. ほとんど全てのことを避けた（ほぼ100％避ける）
25. ここ1週間、自分の表情に関する心配が原因で、以下のリストにある行動をしましたか？実際に行ったことがあるものをすべて選んでください（複数回答あり）
    1. 何かで顔を隠す（自分の手や髪、メガネ、マスクなどを使って隠す）
    2. 他の人と向かい合わせにならないようにする
    3. 表情をつくる
    4. 対面しているときに顔をそらす
    5. 他人の目を気にする / 他人の反応を確認する（他人が顔をそむけたり、自分を避けたり、自分について小声で話していたり、不快そうな態度をしていないか等を確認する）
    6. 目立たない位置にいるようにする（後ろの方や部屋の隅にいる）
    7. 自分が他人からどう見られているのかを想像する
    8. 信頼できる人（家族や友人）に自分の表情が変ではないか質問をして確認する
    9. 表情を隠すための化粧品やアクセサリーなどを買う
    10. 表情を改善するための情報を集める（本や記事を読む / ネット等で調べる）
    11. その他（　　　　　　　　　　　　　　　　　　　　　　　　）
    12. 特に何もしない *この回答を選択した場合、20－21の回答は全て0点となり、22の質問（容姿）へスキップする
26. 上記のリストで選択した行動を、ここ1週間、どのくらいの頻度で行いましたか？（上記にて複数の行動を選択した場合は、総合的にみて、それらの行動をどのくらい行うのかを答えてください）
27. ほとんど行わなかった
28. すこし行った
29. ときどき行った
30. たびたび行った
31. とても頻繁に行った
32. 上記のリストで選択した行動は、ここ1週間、どのくらい社会生活（例： 仕事、学業、他人との交流など）の妨げになりましたか？（複数の行動を選択した場合は、総合的にみて、それらの行動がどのくらい妨げになっているのかを答えてください）
33. 全く妨げになっていなかった
34. すこし妨げになった
35. ある程度の妨げになった
36. けっこう妨げになった
37. かなり妨げになった

自分の外見（容姿）に関する心配ついて

以下の質問では、ここ1週間程度における、あなたの外見の欠点に関する心配について伺います。外見の欠点とは、自分の顔や体型などに対して、あなた自身が「醜い、美しくない、劣っている」と感じている部分のことです。

1. ここ1週間、「自分の外見の欠点が他の人に嫌な感じを与えている」という心配について、どのくらいの頻度で考えましたか？
2. 全く考えなかった　*ここで0と回答した場合、23－28の回答は全て0点となる
3. すこし考えた
4. ときどき考えた
5. たびたび考えた
6. とても頻繁に考えた
7. 「自分の外見の欠点が他の人に嫌な感じを与えている」という考えが事実である（実際に嫌な感じを与えている）と、どのくらい思いますか？
8. 事実ではないと思う（その考えが事実である可能性はほぼ0％）
9. すこしは事実だと思う（10～30％程度）
10. 半信半疑（40～60％程度）
11. おおよそ事実だと思う（70～90％程度）
12. 私の臭いは実際に他人に嫌な感じを与えていると確信する（ほぼ100％）
13. 「自分の外見の欠点が他の人に嫌な感じを与えている」という考えによって、どのくらい、あなたは苦痛を感じますか？
14. 全くない
15. すこし苦痛
16. ある程度苦痛
17. けっこう苦痛
18. とても苦痛
19. ここ1週間、自分の外見の欠点に関する心配が原因で、何かをしたり、どこかに出かけたり、人と関わる状況を避けたりすることがありましたか？
20. 全くない（避ける確率はほぼ0％）
21. すこし避けた（10－30％程度）
22. ときどき避けた（40－60％程度）
23. たびたび避けた（70－90％程度）
24. ほとんど全てのことを避けた（ほぼ100％避ける）
25. ここ1週間、自分の外見の欠点に関する心配が原因で、以下のリストにある行動をしましたか？実際に行ったことがあるものをすべて選んでください（複数回答あり）
    1. 鏡で自分の外見を細かく確認する
    2. 入念に身なりを整える（何度も化粧したり、ヘアースタイルを整えたり、毛を抜いたり、髭を剃ったりする）
    3. 他人の外見を細かくみて自分と比較する
    4. 外見の気になる部分を隠す（洋服や帽子で隠す、日焼けや化粧で隠す、髪やひげ等で隠す、手で覆って隠す、マスクをする、など）
    5. 外見の気になる部位を触る / 測定する（顔の輪郭、吹き出物、傷、しわ、足、お腹、体の脂肪など）
    6. 他人の目を気にする / 他人の反応を確認する（他人が顔をそむけたり、自分を避けたり、自分の外見について小声で話していたり、不快そうな態度をしていないか等を確認する）
    7. 自分が他人からどう見られているのかを想像する
    8. 信頼できる人（家族や友人）に自分の外見が変ではないか質問をして確認する
    9. 外見の欠点を改善するために異常な食生活や過度なダイエットをする
    10. 外見の欠点を改善するために運動をする
    11. 美容商品などを衝動的に購入する
    12. 外科的な施術（美容整形や歯科治療）を受ける / その計画をたてる
    13. 外見の欠点を改善するための情報を集める（本や記事を読む / ネット等で調べる）
    14. 目立たない位置にいるようにする（後ろの方や部屋の隅にいる）
    15. その他（　　　　　　　　　　　　　　　　　　　　　　　　）
    16. 特に何もしない *この回答を選択した場合、27－28の回答は全て0点となる
26. 上記のリストで選択した行動を、ここ1週間、どのくらいの頻度で行いましたか？（上記にて複数の行動を選択した場合は、総合的にみて、それらの行動をどのくらい行うのかを答えてください）
27. ほとんど行わなかった
28. すこし行った
29. ときどき行った
30. たびたび行った
31. とても頻繁に行った
32. 上記のリストで選択した行動は、ここ1週間、どのくらい社会生活（例： 仕事、学業、他人との交流など）の妨げになりましたか？（複数の行動を選択した場合は、総合的にみて、それらの行動がどのくらい妨げになっているのかを答えてください）
33. 全く妨げになっていなかった
34. すこし妨げになった
35. ある程度の妨げになった
36. けっこう妨げになった
37. かなり妨げになった

**採点方法:** 4つの下位尺度の点数と総合スコアを算出できる
— **自己臭:** 項目1，2，3，4，6，7の点数の合計
— **視線:** 項目8，9，10，11，13，14の点数の合計
— **表情:** 項目15，16，17，18，20，21の点数の合計
— 容姿**:** 項目22，23，24，25，27，28の点数の合計
— **総合スコア:** 上記4つの下位尺度の点数の合計
